# Evaluation of the Synergistic Effects of Mineral and Postbiotics Mixtures in Ameliorating Atopic Dermatitis in the NC/Nga Mouse Model

**DOI:** 10.4014/jmb.2511.11018

**Published:** 2026-01-26

**Authors:** Yuseong Jang, Hyeon-Gi Paik, Jihye Choi, Hwal Choi, Jong-Sam Park, Myoung-Hak Kang, Sokho Kim, Jungkee Kwon

**Affiliations:** 1 Department of Laboratory Animal Medicine, College of Veterinary Medicine, Jeonbuk National University, Iksan-si 54596, Republic of Korea; 2 Alldayorganic Co., Ltd., Gimpo-si 10009, Republic of Korea; 3 School of Liberal Studies, Regulatory Science Major, Kunsan National University, Gunsan-si 54150, Republic of Korea

**Keywords:** Atopic dermatitis (AD), Mineral, Postbiotics, Th1/Th2 immune balance

## Abstract

Atopic dermatitis (AD) is a chronic inflammatory skin condition influenced by immune dysregulation. This study aimed to evaluate the effects of a mineral-postbiotic (*Lactobacillus plantarum* VIOAP03 and *Lactobacillus fermentum* VIMPP04) mixture to alleviate AD symptoms in the NC/Nga mouse model. AD was induced in NC/Nga mice using HDM ointment throughout experiment. After inducing AD skin lesions, mice were orally administered ED mineral powder, postbiotics, and a mixture of ED mineral powder and postbiotics. Key parameters measured included dermatitis score, transepidermal water loss (TEWL), and scratching behavior. To assess the effects, serum analyses (IgE, histamine, IgG1, IgG2a) and quantitative reverse transcription polymerase chain reaction (qRT-PCR) were used to quantify Th1/Th2 cytokine responses in dorsal skin tissues. Histological analyses (Hematoxylin & Eosin, Toluidine Blue staining) were performed to assess the dermal thickness and the count of mast cells in the dorsal skin tissues. The mixture group exhibited the most significant improvements in dermatitis score, TEWL, and reduced scratching behavior. Histological analyses showed a decrease in mast cells, along with reduced epidermal thickness, in the mixture group compared to the negative control group. Through the mixture group serum analyses and qRT-PCR, inflammatory markers were downregulated compared to the negative control group, while Th1/Th2 cytokine balance shifted towards reduced Th2 dominance. The combined administration of mineral-postbiotics mixture showed synergistic effects in alleviating AD symptoms by modulating Th1/Th2 cytokine responses. These findings highlight the potential of this combination as a novel therapeutic approach for managing atopic dermatitis.

## Introduction

Atopic dermatitis (AD) is a chronic, relapsing inflammatory skin disease characterized by intense pruritus, xerosis (dry skin), and erythema [1]. AD significantly impairs quality of life, causing sleep disturbances and psychological distress [2, 3]. Since industrialization, the prevalence of AD has steadily increased, with recent estimates showing rates of 16.3% in children aged 0-5 years, 9.4% in those aged 6-12 years, and 9.3% in adults [4-6]. Early intervention in AD is essential, as it is often the first manifestation of the atopic march, which refers to the progression from AD to food allergies, asthma, and allergic rhinitis [7].

The pathogenesis of AD involves immune dysregulation, particularly an imbalance between Th1 and Th2 responses, which normally mutually regulate each other to maintain immune homeostasis [8, 9]. However, when Th2-mediated immunity is hyperactivated, excessive production of Th2 cytokines, such as IL-4 and IL-13, occurs. These cytokines stimulate the production of IgG1 and IgE, thereby leading to mast cell activation and the release of histamine [10]. Activated mast cells amplify the inflammatory response by releasing various mediators, including proteases, cytokines, and chemokines, thereby promoting the recruitment of immune cells and sustaining chronic inflammation [11]. Moreover, Th2 cytokines suppress Th1-associated cytokine production such as IFN-γ, further disrupting the Th1/Th2 balance [12]. The resulting Th2-driven immune environment contributes not only to acute inflammation but also to chronic inflammatory diseases such as AD [13, 14].

Current treatments for AD, including topical corticosteroids (TCS), calcineurin inhibitors, and systemic immunosuppressants, target chronic inflammation and immune dysregulation [15-17]. However, the chronicity of AD limits the long-term use of these drugs, leading to their high cost and potential adverse effects. Prolonged use of TCS is associated with adverse skin atrophy, telangiectasia, and potential adrenal suppression. Long-term treatment with calcineurin inhibitors has been reported to be associated with nephrotoxicity and hypertension [18]. In addition, Janus kinase (JAK) inhibitors have been associated with an increased risk of herpes zoster and thrombocytopenia [19, 20]. Consequently, there is growing interest in bioactive compounds derived from natural sources as potential therapeutic alternatives for AD.

The International Scientific Association for Probiotics and Prebiotics (ISAPP) defines postbiotics as preparations of inanimate microorganisms and/or their components that confer a health benefit on the host [21]. Unlike probiotics, postbiotics are non-viable, which reduces the risk of infection and improves storage stability [22]. Postbiotics have been reported to exert various biological activities, including anti-inflammatory, antioxidant, and immunomodulatory effects [23, 24].

Minerals are essential micronutrients that support key physiological functions, including skeletal health and immune regulation, and are involved in numerous disease-related processes [25, 26]. Specifically, zinc and magnesium have been reported to reduce oxidative stress and modulate immune responses [27, 28]. These functions highlight their importance in disease prevention and the maintenance of overall health. Given their broad physiological roles and bioactive properties, minerals represent promising bioactive agents for enhancing host defense mechanisms and promoting systemic health.

While individual benefits of postbiotics and minerals have been reported, studies examining their co-application remain limited. Given the anti-inflammatory effects of both postbiotics and specific minerals such as zinc and magnesium, their combination may offer synergistic therapeutic benefits in AD. This approach holds promise as a novel anti-allergic strategy for managing the progression of atopic dermatitis. Therefore, this study evaluated the therapeutic potential of a mineral-postbiotic mixture in AD by assessing clinical, biochemical, and immunological parameters, and explored its potential as a platform for combinations with other natural bioactives.

## Materials and Methods

### Composition of ED Mineral Powder

Electrodeposition (ED) mineral powder was provided by the Jeju Seawater Support Center in Jeju Technopark (Republic of Korea). The mineral composition of the ED mineral powder was determined using atomic absorption spectrometry (AAS) and inductively coupled plasma mass spectrometry (ICP-MS). Detailed composition data are shown in Table 1.

### Preparation of Postbiotics

Heat-killed postbiotics were provided by Vitech Co. Ltd., (Republic of Korea) and prepared as follows. *Lactobacillus fermentum* (*L. fermentum*) VIMPP04 was first cultured in standard medium for 8 h as a pre-culture after an 18 h seed culture in flasks, followed by injection into production medium for 12 h of main fermentation. *Lactobacillus plantarum* (*L. plantarum*) VIOAP03 was subjected to an 18 h seed culture in flasks, followed by a 24 h main culture in a medium supplemented with *Phellinus linteus* (*P. linteus*; Berkeley & M.A. Curtis) extract. The concentrated fermentation broth of *L. fermentum* VIMPP04 was combined with the fermented solution of *L. plantarum* VIOAP03 to produce a combined fermentation mixture. This mixture was inactivated using a two-step tyndallization process (step 1: 110°C for 15 min; step 2: 90°C for 30 min). The heat-inactivated mixture was then freeze-dried using a lyophilizer to obtain the final postbiotic powder.

### Animals

Six-week-old male NC/Nga (*n* = 25) mice were purchased from Central Lab Animal, Inc. (Republic of Korea) and acclimatized for one week before the experiment. Mice were fed standard rodent chow (Damool Science, Republic of Korea) and purified water. Animals were housed in individually ventilated cages (IVCs) under conventional housing conditions (temperature, 22 ± 2°C; humidity, 50 ± 10%; 12 h light/dark cycle). All animal experiments were approved by the Institutional Animal Care and Use Committee (IACUC) of Jeonbuk National University, Republic of Korea (JBNU NON2024-212).

### Oral Administration of Samples and Induction of AD-Like Skin Lesions

Mice were assigned to five groups (*n* = 5 per group) using randomization and blinding procedures: normal control (NOR, distilled water); negative control (CON, distilled water); mineral (ED mineral powder 620 mg/kg); postbiotics (1 × 10^11^ cells/kg); mixture (ED mineral powder 620 mg/kg + postbiotics 1 × 10^11^ cells/kg). To disrupt the skin barrier and induce AD-like symptoms, dorsal hair was removed using a hair clipper, followed by the topical application of 150 μl of 4% sodium dodecyl sulfate (SDS) solution to the exposed skin. After 2-3 h, 100 mg of house dust mite (HDM) ointment (*Dermatophagoides farinae*, Biostir-AD; Biostir Inc., Japan) was applied to the dorsal skin twice weekly (on Mondays and Thursdays) throughout the experimental period, except in the NOR group. This treatment continued until the end of the experiment. Following a two-week induction phase, assigned treatments were orally administered once daily from weeks 3 to 6. At the end of the experiment, mice were anesthetized with isoflurane (Hana Pharm. Co., Ltd., Republic of Korea) and blood samples were collected from the retro-orbital venous plexus. After the mice were euthanized using isoflurane, samples of the spleen and dorsal skin were collected.

### Assessment of AD-Like Skin Lesion Severity and Scratching Behavior

Skin lesion severity and scratching behavior were assessed weekly from week 3 to the end of the experiment. Severity was evaluated using a modified SCORAD (SCORing Atopic Dermatitis) index excluding subjective symptoms [29]. Four clinical parameters – erythema/hemorrhage, edema, excoriation/erosion, scaling/dryness – were each scored from a 0-3 scale (0, none; 1, mild; 2, moderate; 3, severe) based on gross visual inspection of the dorsal skin. Scoring was performed according to Biostir-AD guidelines (Biostir Inc., Japan) and was applied consistently throughout the study. The total dermatitis score was calculated by summing the scores of the four parameters, yielding a total score ranging from 0 to 12. All assessments were conducted by a blinded evaluator.

Scratching behavior was assessed using a method modified from Lee *et al*. [30]. Mice were individually placed in observation cages for a 30 min acclimation period before evaluation. Following HDM ointment application, scratching events, defined as repetitive hind paw movements contacting the dorsal skin [31], were counted for 10 min. Grooming behaviors, including paw licking, bringing the hind paws to the mouth, or placing them on the cage floor, were excluded [32].

### Assessment of Transepidermal Water Loss (TEWL)

The transepidermal water loss (TEWL) on the dorsal skin was evaluated weekly, starting in week 3, until the end of the experiment, using the GP Skin Barrier Light (Gpskin, Republic of Korea) to assess skin barrier function. Measurements were conducted under controlled environmental conditions (22 ± 2°C, 50 ± 5% relative humidity) at a consistent dorsal site, with the probe positioned perpendicular to the skin surface. Each assessment lasted roughly 10 sec and was repeated three times per mouse, with the average value being used for further analysis. The GP Skin Barrier Light has been validated against gold-standard devices such as AquaFlux and Corneometer, showing high correlation and reproducibility in both clinical and experimental settings [33, 34].

### Analyses of Serum Immunological Marker and Inflammatory Cytokines

At the end of the experiment, blood samples were centrifuged at 800 × *g* for 15 min. The supernatant serum was stored at -80°C until analysis. Serum levels of immunoglobulin E (IgE, EMIGHE, Invitrogen, USA), histamine (MOEB2524, AssayGenie, Ireland), immunoglobulin G1 (IgG1, 88-50410, Invitrogen), and immunoglobulin G2a (IgG2a, 88-50420, Invitrogen) were quantified using enzyme-linked immunosorbent assay (ELISA) kits according to the manufacturer’s instructions. Absorbance was read at 450 nm using a microplate reader (BioTek Instruments, USA).

### Splenocyte Isolation and Cell Proliferation

At the end of the experiment, spleens were removed and placed in Hank’s Balanced Salt Solution (HBSS; Gibco BRL, USA). Tissues were homogenized through a 40-μm cell strainer (Corning, USA) to obtain single-cell suspensions, which were layered onto Histopaque (Sigma-Aldrich, USA) and centrifuged at 280 × *g* for 3 min to isolate splenocytes via density gradient separation. The splenocyte layer was collected, washed, and centrifuged at 500 × *g* for 5 min. Cells were resuspended and seeded into 96-well plates at a density of 1 × 10^6^ cells/well in RPMI-1640 medium supplemented with 10% heat-inactivated fetal bovine serum (FBS) and 1% penicillin/streptomycin. Splenocyte proliferation was assessed by stimulating cells with concanavalin A (Con A, 5 μg/ml) or lipopolysaccharide (LPS, 5 μg/ml) for 48 h at 37°C in 5% CO_2_. Con A, a plant-derived mitogen, activates T lymphocytes, whereas LPS, a Gram-negative bacterial component, stimulates B cell proliferation. Cell viability was determined using the WST-8 assay kit (QM1000; BIOMAX, Republic of Korea), following the manufacturer’s instructions, and absorbance was read at 450 nm using a microplate reader (BioTek Instruments, USA).

### RNA Isolation and Quantitative Reverse Transcription Polymerase Chain Reaction (qRT-PCR) Analyses

Total RNA was extracted from the dorsal skin tissue using a RNeasy Mini Kit (74104, Qiagen, USA). The purity and concentration of RNA were determined using a NanoDrop spectrophotometer (BioSpec-nano, Shimadzu, Japan). The isolated RNA was reverse-transcribed into cDNA using ReverTra Ace qPCR RT Master Mix with gDNA Remover kit (FSQ-301, TOYOBO, Japan). RT-qPCR was performed with the BioFACT 2X Real-Time PCR Master Mix, including SYBR Green I and High ROX reference dye (DQ385-40h, BIOFACT, Republic of Korea) on a StepOne Real-Time PCR System (Applied Biosystems, USA) using a three-step cycling protocol. Gene expression was quantified using the 2^-(ΔΔCt)^ method, with GAPDH as the internal control. Primer sequences are shown in Table 2.

### Histological Analyses

After the mice were sacrificed, dorsal skin tissue was collected and fixed in 4% paraformaldehyde for 24 h, dehydrated through a graded ethanol series (70-100%), cleared in xylene, and infiltrated with paraffin wax at 60°C. Tissues were embedded in paraffin blocks and sectioned at a thickness of 5 μm using a microtome. For hematoxylin and eosin (H&E) staining, sections were stained with hematoxylin for 3 min, differentiated in 1% acid alcohol, and counterstained with eosin for 1 min to assess epidermal thickness.

To detect mast cells, sections were stained with 0.1% toluidine blue (TB, pH 2.0-2.5) for 3 min and differentiated in 95% ethanol. Epidermal thickness was measured from the basal layer to the lower margin of the stratum corneum, and mast cells in the dermis were counted using a light microscope (Zeiss, Germany) at 200 × magnification across five randomly selected fields per sample. All histological assessments were performed by two independent observers in a blinded manner to ensure objectivity and reliability.

### Statistical Analyses

Data are expressed as mean ± standard deviation (SD). Statistical analyses were conducted using one-way analysis of variance (ANOVA) followed by Tukey’s multiple range test. All analyses were performed using GraphPad Prism version 9.5 (GraphPad Software, USA), with statistical significance set at *p* < 0.05.

## Results

### Mixture of Mineral and Postbiotics Ameliorates AD-Like Skin Lesions on HDM-Induced NC/Nga Mice

**Fig. 1:**
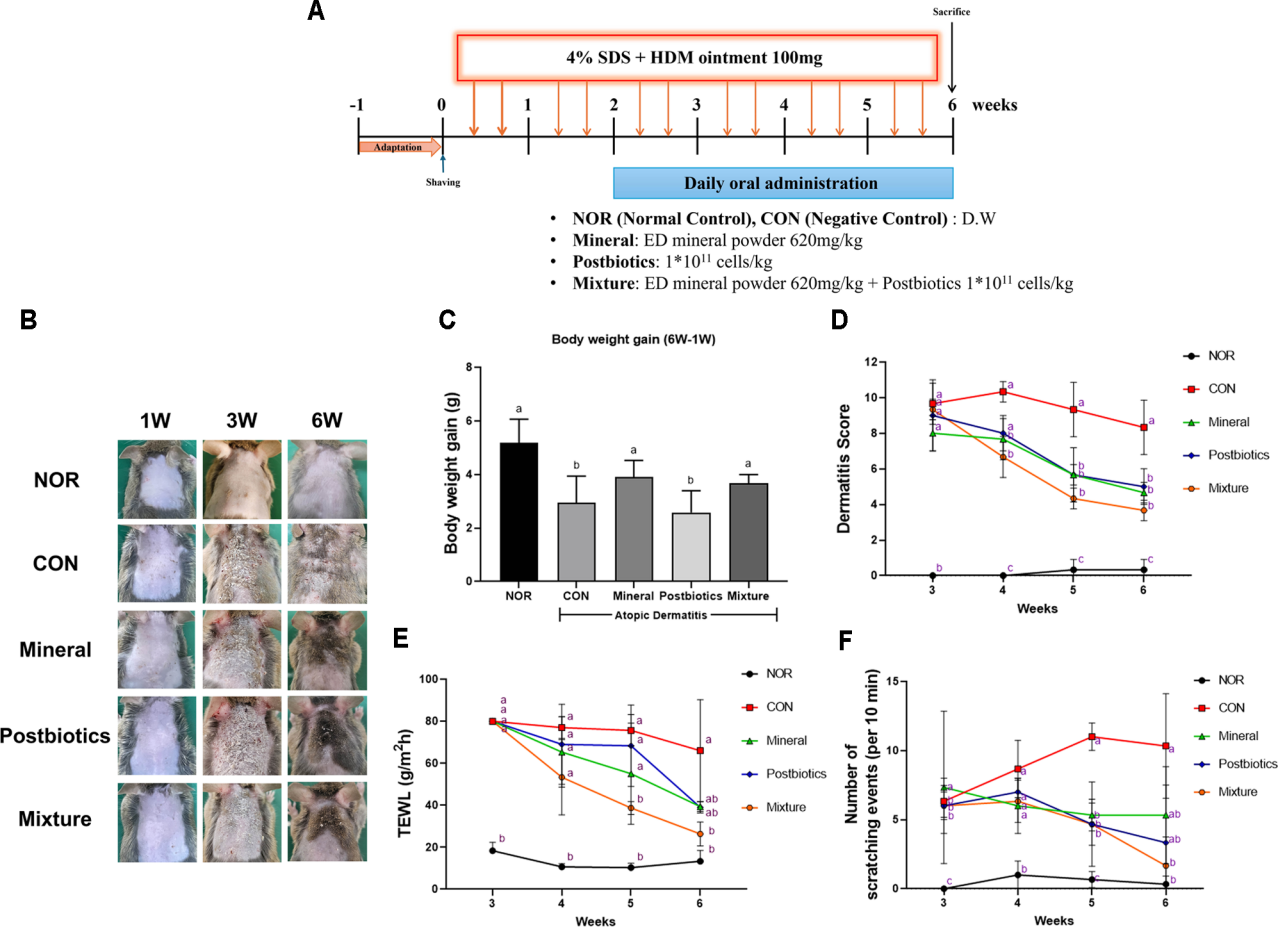
Synergistic effects of combined mineral and postbiotics on improvement of clinical features caused by HDM-induced AD-like skin lesions in NC/Nga mice. (**A**) Experiment design. (**B**) Representative images of dorsal skin at 1, 3, and 6 weeks of the experimental period. (**C**) Changes in body weight throughout the experimental period. (**D**) Skin severity score based on four parameters: erythema/hemorrhage, edema, excoriation/erosion, scaling/dryness. (**E**) Transepidermal water loss (TEWL). (**F**) Scratching behavior was measured over a 10 min observation period. Data are expressed as mean ± SD (*n* = 5). a-c Different labels were significantly different at *p* < 0.05 between treatments, as determined by one-way ANOVA followed by Tukey’s post hoc test.

Animal experiments were conducted according to the timeline shown in Fig. 1A to evaluate the therapeutic potential of mineral, postbiotics, and their combination (mixture) on HDM-induced AD-like skin lesions in NC/Nga mice. After 3 weeks of treatment, evident AD symptoms, including erythema, edema, excoriation, and scaling, were observed in all groups except the NOR group. By week 6, all treated groups showed visible improvements in dorsal skin lesions and recovered body weight, with the mixture group demonstrating the most pronounced effect compared with the CON group (Fig. 1B, C; *p* < 0.05). Dermatitis severity scores (Fig. 1D) exceeded 8 points in all experimental groups by week 3. However, by week 4, the mixture group exhibited the most substantial reduction in dermatitis scores, which persisted through week 6 (*p* < 0.05). The mineral and postbiotics groups also showed progressive improvements during this period. TEWL, initially elevated (~80 g/m^2^/h) at week 3, declined progressively in all treatment groups. By week 6, TEWL levels in the mixture group approximated normal values (Fig. 1E). This reduction in TEWL was consistent with the observed decrease in scratching frequency and alleviation of dermatitis severity. By week 6, scratching behavior was significantly reduced in the mixture group compared with the CON group (Fig. 1F; *p* < 0.05), indicating the effective alleviation of pruritic symptoms.

### Mixture of Mineral and Postbiotics Suppressed Hyperplasia of Mast Cells on HDM-Induced NC/Nga Mice

**Fig. 2:**
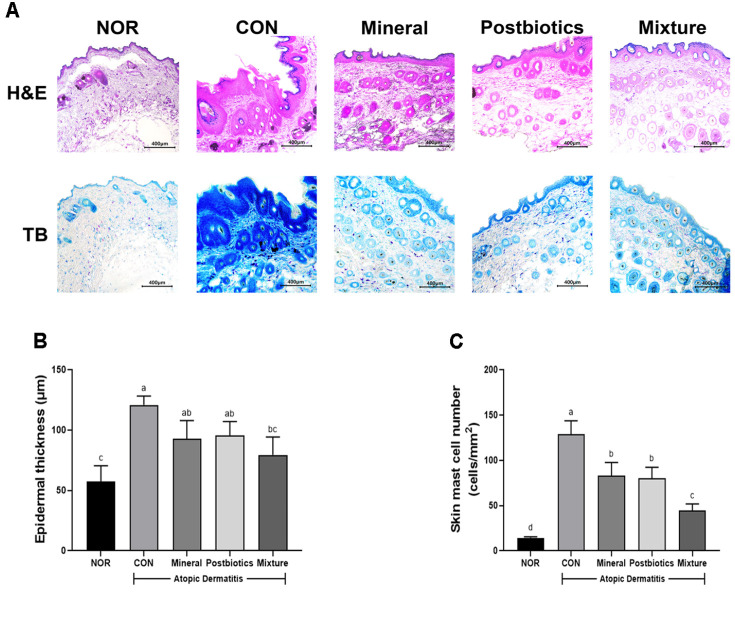
Synergistic effects of combined mineral and postbiotics on epidermal thickness and infiltration of mast cells of HDM-induced ADlike skin lesions in NC/Nga mice. (**A**) Representative images of dorsal skin stained with hematoxylin and eosin (H&E, above) and toluidine blue (TB, below). Images were observed under a microscope at 200× magnification. Scale bars = 400 μm. (**B**) Thickness of epidermis in H&E-stained section. (**C**) Number of skin mast cells in TB-stained section. Data are expressed as mean ± SD (*n* = 5). a-d Different labels were significantly different at *p* < 0.05 between treatments, as determined by one-way ANOVA followed by Tukey’s post hoc test.

To evaluate epidermal thickness and mast cell infiltration, sections of dorsal skin were stained with hematoxylin and eosin (H&E) and toluidine blue (TB) (Fig. 2A). The CON group exhibited significantly increased epidermal thickness and elevated mast cell counts compared with the NOR group (*p* < 0.05), indicating pronounced inflammation (Fig. 2B and 2C). Both the mineral and postbiotics groups showed moderate reductions, whereas the mixture group exhibited a notable improvement among the treatment groups. Epidermal thickness and mast cell infiltration in the mixture group were significantly reduced compared with the CON group (*p* < 0.05). These histological improvements correlated closely with the functional outcomes, such as reduced dermatitis scores and improved skin barrier function, suggesting that the combined treatment effectively mitigates inflammation and pruritus through decreased mast cell activation.

### Mixture of Mineral and Postbiotics Regulated AD-Related Allergic Response Markers on HDM-Induced NC/Nga Mice

**Fig. 3:**
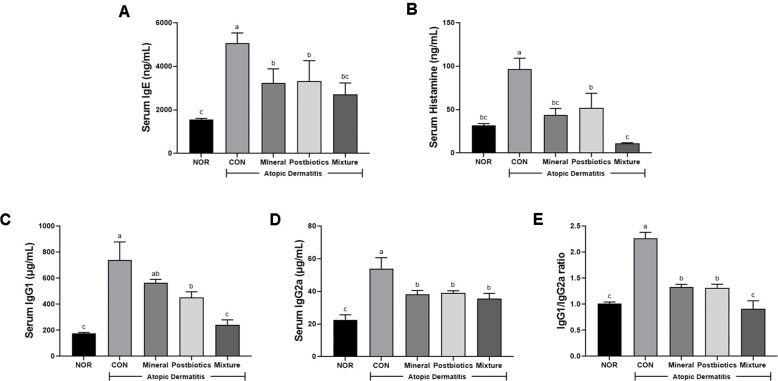
Synergistic effects of combined mineral and postbiotics on serum levels of (A) IgE, (B) Histamine, (C) IgG1, (D) IgG2a, and (E) the IgG1/IgG2a ratio of HDM-induced AD-like skin lesions in NC/Nga mice. The IgG1/IgG2a ratio was normalized to the normal group as a reference. Serum samples were collected on the final day of the experiment and analyzed using ELISA kits. Data are expressed as mean ± SD (*n* = 5). a-c Different labels were significantly different at *p* < 0.05 between treatments, as determined by one-way ANOVA followed by Tukey’s post hoc test.

As shown in Fig. 3A, serum IgE levels were higher in the CON group than in the NOR group (*p* < 0.05), while treatment with the mixture significantly lowered IgE levels compared with the CON group (*p* < 0.05). Similarly, serum histamine levels were significantly elevated in the CON group compared with the NOR group, whereas the mixture group was significantly reduced in the treatment groups (Fig. 3B; *p* < 0.05). Serum IgG1 levels in the mixture group were lower compared with the CON group (Fig. 3C; *p* < 0.05). Serum IgG2a levels showed the highest in the CON group, and the elevated levels were significantly reduced in all treatment groups (Fig. 3D). Notably, the IgG1/IgG2a ratio was significantly higher in the CON group (2.26 ± 0.09) compared with the NOR group (1.01 ± 0.03). In contrast, the ratio was markedly reduced in the mixture group (0.92 ± 0.12), representing a 59.6% decrease compared with the CON group (*p* < 0.05; Fig. 3E). These results indicate that the treatment of the mixture effectively modulates allergic inflammation by suppressing Th2-driven mechanisms and restoring Th1/Th2 immune balance.

### Mixture of Mineral and Postbiotics Modulated Adaptive Immune Cell Proliferation in HDM-Induced NC/Nga Mice

**Fig. 4:**
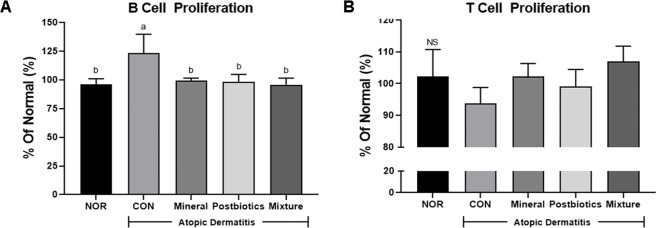
Combined effects of mineral and postbiotics on B and T cell proliferation in splenocytes induced by HDM-induced AD-like skin lesions in NC/Nga mice. (**A**) B cell proliferation following stimulation with lipopolysaccharide (LPS, 5 μg/ml). (**B**) T cell proliferation following stimulation with concanavalin A (Con A, 5 μg/ml). Data are expressed as mean ± SD (*n* = 5). a-b Different labels were significantly different at *p* < 0.05 between treatments, as determined by one-way ANOVA followed by Tukey’s post hoc test. ^NS^ This term indicates no significant difference among groups.

To evaluate the immunomodulatory effects of treatments on adaptive immune function, B cell and T cell proliferation assays were conducted using isolated splenocytes from each group. As shown in Fig. 4A, proliferation of B cell was was significantly higher in the CON group compared with the NOR group (*p* < 0.05). In contrast, the treatment groups showed significantly decreased proliferation compared with the CON group (*p* < 0.05). T cell proliferation exhibited no statistically significant differences among groups (Fig. 4B). However, the CON group (93.75 ± 2.89) exhibited slightly lower T cell proliferation levels than the NOR group (102.17 ± 4.93), whereas the mixture group (106.93 ± 2.80) showed a slightly higher increase than the NOR group. Together, these findings suggest that the mixture exerts balanced immunomodulatory effects by suppressing exaggerated humoral responses and potentially supporting cellular immune activation.

### Mixture of Mineral and Postbiotics Suppressed Th2-Related Chemokine Expression in HDM-Induced NC/Nga Mice

**Fig. 5:**
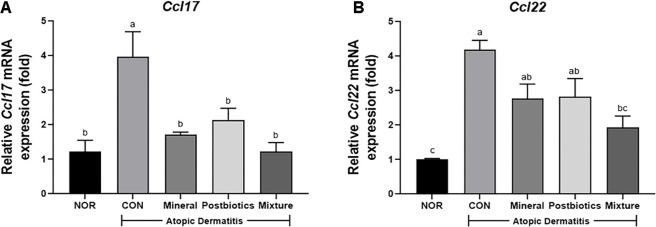
Synergistic effects of combined mineral and postbiotics on gene expression of Th2-mediated chemokines in HDM-induced AD-like skin lesions in NC/Nga mice. (**A**) Fold change in activation-regulated chemokine (*Ccl17*) mRNA expression relative to the normal group. (**B**) Fold change in macrophage-derived chemokine (*Ccl22*) mRNA relative to the normal group. Data are expressed as mean ± SD (*n* = 5). a-b Different labels were significantly different at *p* < 0.05 between treatments, as determined by one-way ANOVA followed by Tukey’s post hoc test.

To investigate the effects of the treatments on Th2 chemokine expression, the mRNA levels of *Ccl17* (thymus and activation-regulated chemokine/TARC) and *Ccl22* (macrophage-derived chemokine/MDC) were measured in dorsal skin tissue. Both chemokines play important roles in attracting and activating Th2 cells and are involved in allergic inflammation. As shown in Fig. 5A, *Ccl17* mRNA expression was significantly higher in the CON group (3.97 ± 0.51) than in the NOR group (1.23 ± 0.22). The mixture group (1.23 ± 0.18) exhibited the lowest mRNA expression among the treatment groups, with levels comparable to those of the NOR group. Similarly, as shown in Fig. 5B, *Ccl22* mRNA expression was significantly downregulated in the mixture group (1.93 ± 0.23) compared with the CON group (4.18 ± 0.20). These reductions in chemokine expression were consistent with previously observed improvements in skin inflammation, serum IgE, and histamine, collectively suggesting effective modulation of Th2-driven allergic responses.

### Mixture of Mineral and Postbiotics Downregulated Th2 Cytokine Expression in HDM-Induced NC/Nga Mice

**Fig. 6:**
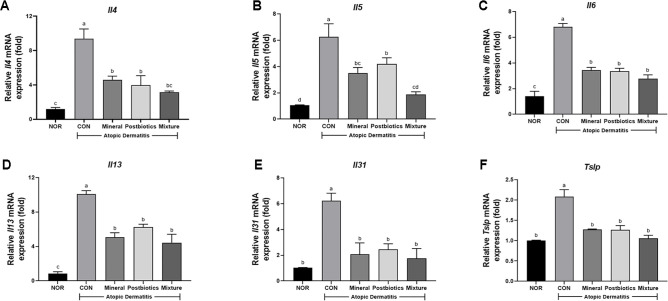
Synergistic effects of combined mineral and postbiotics on gene expression of Th2 cytokines in HDM-induced AD-like skin lesions in NC/Nga mice. Fold change in (**A**) *Il4*, (**B**) *Il5*, (**C**) *Il6*, (**D**) *Il13*, (**E**) *Il31*, and (**F**) thymic stromal lymphopoietin (*Tslp*) mRNA expression relative to the normal group. Data are expressed as mean ± SD (*n* = 5). a-d Different labels were significantly different at *p* < 0.05 between treatments, as determined by one-way ANOVA followed by Tukey’s post hoc test.

To assess the effects of treatments on Th2-mediated inflammation, mRNA expression levels of *Il4*, *Il5*, *Il6*, *Il13*, *Il31*, and thymic stromal lymphopoietin (*Tslp*) were measured in dorsal skin tissue. The expression levels of Th2-mediated cytokines such as *Il4*, *Il5*, *Il6*, *Il13*, *Il31*, and *Tslp* were significantly increased in the CON group compared with the NOR group (Fig. 6; *p* < 0.05). However, the mixture group was downregulated in these Th2-mediated cytokine expressions, showing reductions in *Il4* (67.3%), *Il5* (72.0%), *Il6* (61.3%), *Il13* (55.9%), *Il31* (71.8%), and *Tslp* (55.9%) compared with the CON group (*p* < 0.05). These findings suggest that the mixture effectively attenuated Th2-mediated cytokine expression in the HDM-induced AD model.

### Mixture of Mineral and Postbiotics Restored Th1 Cytokine Expression Suppressed by HDM-Induced NC/Nga Mice

**Fig. 7:**
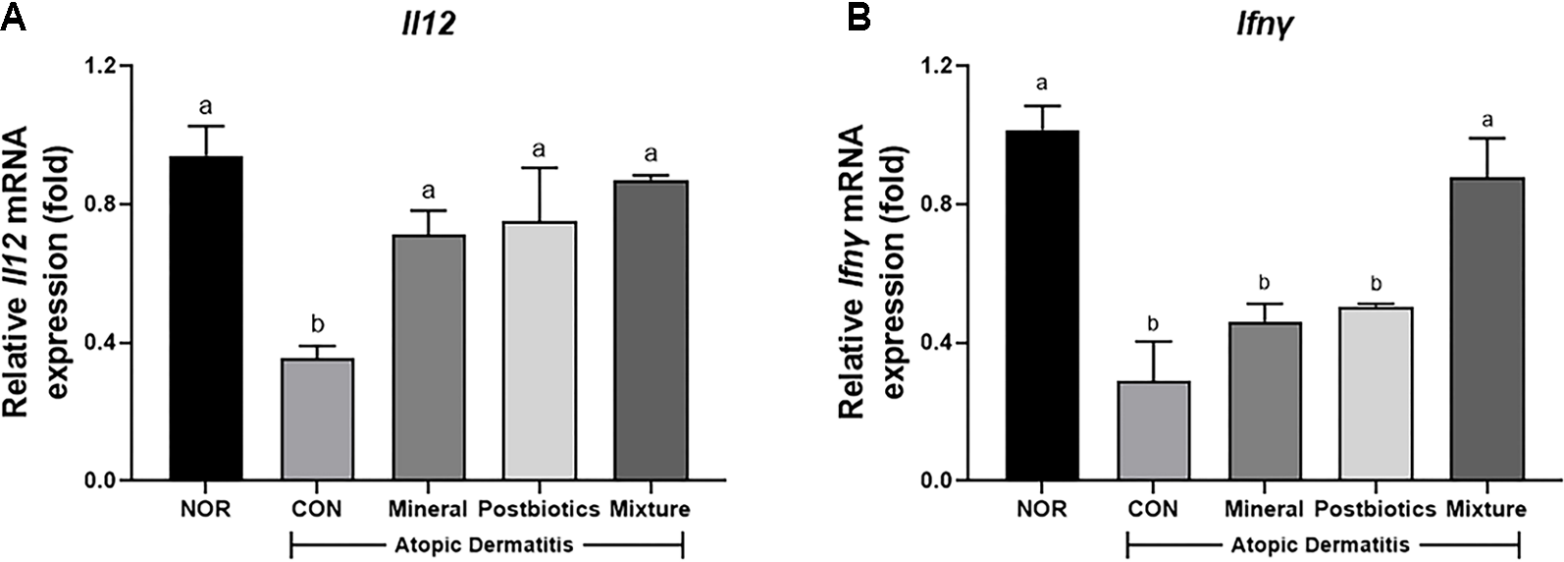
Synergistic effects of combined mineral and postbiotics on gene expression of Th1 cytokines in HDM-induced AD-like skin lesions in NC/Nga mice. (**A**) Fold change in *Il12* mRNA expression relative to the normal group. (**B**) Fold change in *Ifnγ* mRNA expression relative to the normal group. Data are expressed as mean ± SD (*n* = 5). a-b Different labels were significantly different at *p* < 0.05 between treatments, as determined by one-way ANOVA followed by Tukey’s post hoc test.

To evaluate Th1 immune responses, mRNA expression levels of *Il12* and *Ifnγ* were measured in dorsal skin tissue (Fig. 7). The CON group exhibited significantly reduced expression of both cytokines compared with the NOR group (*p* < 0.05). Notably, the mixture group upregulated expression of *Ifnγ* and *Il12*, showing 67.0% and 59.2% higher levels, respectively, compared with the CON group (*p* < 0.05). These findings suggest that the mixture treatment contributes to restoring Th1/Th2 immune responses toward a state of homeostasis.

## Discussion

Damage to skin barrier proteins such as filaggrin, involucrin, and loricrin due to a repetitive itch-scratch cycle is a well-known characteristic of AD and plays a central role in disease progression [35, 36]. Continuous scratching disrupts the skin barrier, facilitates the invasion of pathogens, and triggers immune responses. In this study, the mixture significantly reduced dermatitis scores (Fig. 1D), TEWL (Fig. 1E), and scratching behavior (Fig. 1F). The reduction in TEWL, along with decreased scratching behavior, indicates that the mixture contributed to the restoration of skin barrier integrity and may lower the risk of secondary microbial infection [37]. These results suggest that the mixture may alleviate scratch-induced skin damage by enhancing the recovery of the skin barrier.

Regulation of IgE and histamine is central to allergic reactions. Previous studies have demonstrated that allergen-sensitized Th2 cells secrete IL-4 and IL-13, which in turn stimulate B cells to produce IgE [38, 39]. The allergen-specific IgE mediates mast cell degranulation, leading to the release of pro-inflammatory mediators such as histamine, leukotrienes, prostaglandin E2, and tryptase [40, 41]. In this study, the mixture group significantly reduced serum IgE and histamine levels (Fig. 3A and 3B) and inhibited mast cell infiltration into dorsal skin tissues (Fig. 2C). The inhibition of mast cell infiltration may have the potential to reduce angiogenesis and ameliorate eczema symptoms [42, 43]. Therefore, these findings suggest that the mixture may mitigate allergic responses by attenuating the activation of inflammatory mediators in the HDM-induced AD model.

Th1/Th2 immune balance is a representative immunological feature in allergic diseases such as AD [44, 45]. AD comprises both acute and chronic phases, in which the acute phase is characterized by predominant Th2 immune activation and the chronic phase involves both Th2- and Th1-mediated responses [35]. Among biomarkers, IgG1 is generally considered an indicator of Th2 immunity, and IgG2a reflects Th1 responses. In this study, the mixture significantly restored the Th1/Th2 balance through regulating the serum IgG1/IgG2a ratio (Fig. 3E). At the gene expression level, Th2 cytokines such as *Il4* and *Il13* were downregulated (Fig. 6), whereas Th1 cytokines were upregulated in dorsal skin tissue (Fig. 7). Therefore, consistent with previous studies, these results indicate that this treatment helps restore the Th1/Th2 balance [46-48]. In summary, the inhibition of Th2 cytokines and inflammatory cell infiltration may lead to a reduction in serum biomarkers such as histamine, which may underlie the restoration of Th1/Th2 balance [49, 50]. This immune-modulating ability highlights the potential of the mixture as a therapeutic strategy for AD and related allergic diseases.

Postbiotics have recently attracted attention for their safety, stability, and pharmacological potential [51, 52]. In this study, we employed postbiotics derived from *P. linteus*, a traditional medicinal fungus. The bioactivity of *P. linteus* is attributed to β-glucans, which are well-known for their anti-inflammatory and immunomodulatory properties [53, 54]. Consistent with these properties, *P. linteus* has been shown to improve SCORAD index and pruritus scores in AD patients and to exert anti-allergic effects by modulating immune responses [55]. Additionally, minerals are essential cofactors for enzymatic reactions and play critical roles in immune defense, thereby forming the basis of many physiological functions [56]. Magnesium and zinc are important for immune defense; for example, both are known to suppress NF-κB signaling and to downregulate pro-inflammatory cytokines, and zinc supports T-cell maturation through thymulin activity [28, 57]. Given that postbiotics enhance immunity and mineral regulate key inflammatory pathways, their combination may provide synergistic immunomodulatory benefits-a possibility that remains underexplored in AD models [43, 58].

While the amelioration of AD-related features observed in the present study, the precise cellular targets and molecular mechanisms underlying the effects of the mineral-postbiotics mixture remain to be further clarified. Future studies employing receptor-deficient models, such as Dectin-1 knockout or Nod1/2 knockout mice, could provide additional insight into how these components contribute to the observed effects. Such approaches may help to identify primary target cell populations, including immune cells and skin-resident epithelial cells, and to better understand how the mineral and postbiotics mixture influences specific AD-associated immune and histological phenotypes. These future investigations would contribute to a more detailed mechanistic interpretation and support the further development of this formulation as an immunomodulatory strategy.

This study demonstrates that the combined administration of postbiotics and mineral not only attenuates clinical and histological features of AD but also supports the restoration of immune balance at both systemic and local levels. The observed synergy between these agents suggests an enhanced immunomodulatory capacity that exceeds the effects of either component alone. By modulating Th1- and Th2-associated immune pathways, this approach may alleviate acute allergic inflammation, prevent the progression to a chronic immune state, and restore immune homeostasis. Collectively, these findings suggest preliminary evidence that the mineral-postbiotics mixture promise as a therapeutic strategy for the early intervention and management of allergic skin diseases.

## Figures and Tables

**Table 1:** Composition of ED mineral powder.

Material	Component (mg/g)	Material	Component (μg/g)
Mg	112.8	Zn	1.8
Na	57.7	Se	<1.0
Ca	29.2	U	<1.0
K	1.2	Mo	<1.0
		Cr	<1.0
		V	<1.0
		Mn	<1.0
		Fe	<1.6
		Ni	<1.0
		Cu	<1.0
		As	<1.0

**Table 2:** Primer sequences

Target Gene		Sequence of PCR Primer (5'-3')
*Il4*	F	ACAGGAGAAGGGACGCCAT
	R	GAAGCCCTACAGACGAGCTCA
*Il5*	F	TCAGCTGTGTCTGGGCCACT
	R	TTATGAGTAGGGACAGCAAGCCTCA
*Il6*	F	TAGTCCTTCCTACCCCAATTTCC
	R	TTGGTCCTTAGCCACTCCTTC
*Il13*	F	CAATTGCAATGCCATCTACAGGAC
	R	CGAAACAGTTGCTTTGTGTAGCTGA
*Il12*	F	CATCGATGAGCTGATGCAGT
	R	CAGATAGCCCATCACCCTGT
*Ifnγ*	F	TCAAGTGGCATAGATGTGGAAGAA
	R	CCATCCTTTTGCCAGTTCCTC
*Il31*	F	TCAGCAGACGAATCAATACAGC
	R	TCGCTCAACACTTTGACTTTCT
*Ccl22*	F	TCTGATGCAGGTCCCTATGGT
	R	TTATGGAGTAGCTTCTTCAC
*Ccl17*	F	CAGGAAGTTGGTGAGCTGGTATA
	R	TTGTGTTCGCCTGTAGTGCATA
*Tslp*	F	CTGTACTGTTAATGACCAGC
	R	TCGTAGATGAAGGCTCT
*Gapdh*	F	CGGCCGCATCTTCTTGTG
	R	CCGACCTTCACCATTTTGTCTAC
